# Cross-dimensional electron-phonon coupling in van der Waals heterostructures

**DOI:** 10.1038/s41467-019-10400-z

**Published:** 2019-06-03

**Authors:** Miao-Ling Lin, Yu Zhou, Jiang-Bin Wu, Xin Cong, Xue-Lu Liu, Jun Zhang, Hai Li, Wang Yao, Ping-Heng Tan

**Affiliations:** 1 State Key Laboratory of Superlattices and Microstructures, Institute of Semiconductors, Chinese Academy of Sciences, 100083 Beijing, China; 2 Center of Materials Science and Optoelectronics Engineering & CAS Center of Excellence in Topological Quantum Computation, University of Chinese Academy of Sciences, 100049 Beijing, China; 3 Key Laboratory of Flexible Electronics and Institute of Advanced Materials, Jiangsu National Synergetic Innovation Center for Advanced Materials, Nanjing Tech University, 30 South Puzhu Road, 211816 Nanjing, China; 4 Beijing Academy of Quantum Information Science, 100193 Beijing, China; 5 Department of Physics and Centre of Theoretical and Computational Physics, University of Hong Kong, Hong Kong, China

**Keywords:** Two-dimensional materials, Electronic properties and materials

## Abstract

The electron-phonon coupling (EPC) in a material is at the frontier of the fundamental research, underlying many quantum behaviors. van der Waals heterostructures (vdWHs) provide an ideal platform to reveal the intrinsic interaction between their electrons and phonons. In particular, the flexible van der Waals stacking of different atomic crystals leads to multiple opportunities to engineer the interlayer phonon modes for EPC. Here, in hBN/WS_2_ vdWH, we report the strong cross-dimensional coupling between the layer-breathing phonons well extended over tens to hundreds of layer thick vdWH and the electrons localized within the few-layer WS_2_ constituent. The strength of such cross-dimensional EPC can be well reproduced by a microscopic picture through the mediation by the interfacial coupling and also the interlayer bond polarizability model in vdWHs. The study on cross-dimensional EPC paves the way to manipulate the interaction between electrons and phonons in various vdWHs by interfacial engineering for possible interesting physical phenomena.

## Abstract (web summary)

The strength of electron-phonon coupling can be directly probed by Raman spectroscopy. Here, the authors use low-frequency Raman spectroscopy to unveil the existence of a strong cross-dimensional coupling between the bulk-like layer-breathing phonons in an hBN/WS2 heterostructure and the electrons localized within its few-layer WS2 constituent.

## Introduction

The coupling between phonons and electrons, one of the fundamental coupling of quasiparticles in solids is the key to many unusual quantum effects in thermodynamics^1^, superconductivity^2,3^, transport^4,5^, and optical phenomena^6,7^. The electron-phonon coupling (EPC) in a bulk material is dictated by its lattice vibrations and electronic band structures, leaving little room for its tunability. In three-dimensional (3D) to two-dimensional (2D) crossover regime, the EPC is reported to be modified and generate numerous fascinating physical effects, such as 2D Ising superconductivity^8^, enhanced electron-phonon scattering rate^9^, and electron coherence in reduced dimensional systems^5^. The emergence of 2D materials (2DMs) has led to the advances in engineering EPC by electronic doping^10–13^ or phonon dimensionality modulation^5,9,14^. The rich possibilities in forming van der Waals heterostructures (vdWHs) by vertically assembling different 2DMs with varieties of choices of components, thickness and interface engineering further provide multiple approaches to manipulate the interactions between the electrons, phonons, and excitons^6,15–23^, thus to modulate their opto-electronic properties^17,22,24–30^. The interlayer coupling in vdWHs allows engineering the dimensionality of both the electrons and phonons. The layer distribution of electron wavefunction is essentially determined by the band alignment, while that of the phonon wavefunction is controlled by the force constants. These separate controls in the spatial extension of electron and phonon wavefunctions allow EPC to be addressed in three distinct regimes: (1) bulk-like EPC between layer-extended electrons and phonons^26^; (2) EPC in the 2D limit between layer-localized electrons and phonons^6,7^; and (3) between layer-localized 2D electrons and layer-extended bulk-like phonons, or vice versa. The last regime can be of particular interest for exploring additional EPC physics but still inaccessible to the community.

In general, the electron-phonon interaction of a material can be directly probed by the peak intensity of a phonon mode in the  Raman spectroscopy. Here, we report the evidence of cross-dimensional electron-phonon interaction between the 3D layer-breathing (LB) phonons in a thick hBN/WS_2_ vdWH up to hundreds of layers and 2D electrons of its few-layer WS_2_ constituent. In contrast to a few LB modes observed in multilayer WS_2_ (MLW), Raman spectra in hBN/WS_2_ vdWHs show a large number of LB modes when the excitation energy (*E*_ex_) is resonant with the C exciton energy (*E*_C_). This observation is attributed to the coupling between layer-localized 2D electrons confined to the few-layer WS_2_ constituents and bulk-like LB phonons extended over the entire vdWHs. This cross-dimensional EPC strength is consistent with the phonon wavefunction projection between the studied layer-extended bulk-like LB modes in hBN/WS_2_ vdWHs and the LB modes in the corresponding standalone WS_2_ flakes that are strongly coupled with the C exciton, which can be further confirmed by the calculated Raman intensity of the LB modes in hBN/WS_2_ vdWHs based on the interlayer bond polarizability model. This electron-phonon interaction is intrinsically different from the previously reported interlayer EPC^6^ between optically silent intralayer hBN phonons and two resonant states in monolayer WSe_2_ based hBN/WSe_2_ vdWHs. This work suggests additional possibilities to manipulate electron-phonon interaction in various vdWHs for exploring unusual quantum phenomena and applications.

## Results

### Enhanced interlayer LB modes in hBN/WS_2_ vdWHs

**Fig. 1:**
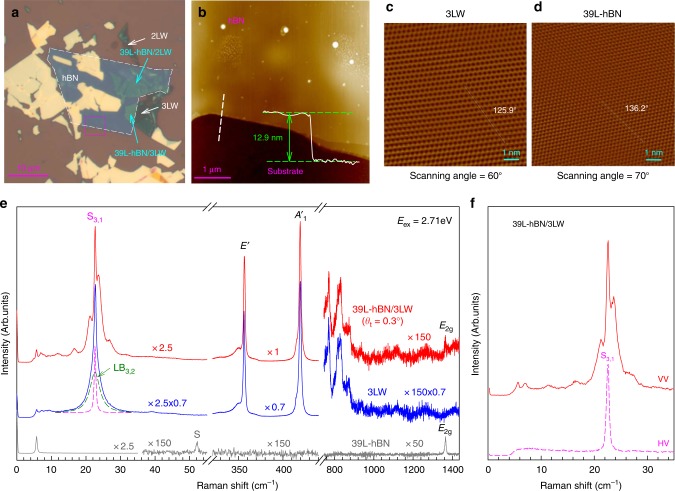
Characterizations and Raman spectra of the 3LW flake and hBN/3LW vdWHs. **a** Optical images of 2LW, 3LW, 39L-hBN/2LW and 39L-hBN/3LW. **b** AFM image of the hBN flake with thickness of 12.9 nm in pink box of **a**. The atomic lattice images of **c** 3LW and **d** 39L-hBN flakes. The dashed white lines in **c**, **d** represent the lattice orientations of 3LW and 39L-hBN are 125.9° and 136.2°, respectively. The scanning angles are 60° and 70° for 3LW and 39L-hBN, respectively. The twist angle *θ*_t_ between the 39L-hBN and 3LW flakes is 0.3°. **e** Raman spectra of 39L-hBN, 3LW and 39L-hBN/3LW. The green dash profile depicts the LB_3,2_ mode in 3LW while the pink dash profile represents the S_3,1_ mode. The spectra are scaled and offset for clarify and the scale factors are shown. **f** The polarized (VV) and depolarized (HV) Raman spectra of 39L-hBN/3LW

hBN/WS_2_ vdWHs with number of total layers, *N* = *m* + *n*, can be formed by transferring the *n*-layer hBN flake (*n*L-hBN) onto *m*-layer WS_2_ flake (*m*LW) or the *m*LW flake onto *n*L-hBN flake (see Methods), named as *n*L-hBN/*m*LW and *m*LW/*n*L-hBN, respectively. Figure 1a shows the hBN/WS_2_ vdWHs, along with 2LW, 3LW, and 39L-hBN flakes (corresponding to 12.9 nm thickness in Fig. 1b). The atomic lattice images of 3LW and 39L-hBN flakes are shown in Fig. 1c, d, respectively. Interlayer EPC in monolayer-WSe_2_/hBN (1L-WSe_2_/hBN) vdWHs can enhance the Raman signal of optically silent high-frequency hBN intralayer phonons through resonantly coupling to the A exciton transition of 1L-WSe_2_^6^. However, the corresponding ZO mode (~820 cm^−1^)^6^ of hBN constituents was not observed in the Raman spectrum of 1LW/hBN vdWHs when *E*_ex_ matches *E*_C_ of 1LW^31,32^ (Supplementary Fig. 1). The case is also true for hBN/3LW vdWHs, as illustrated in Fig. 1e. The calibrated peak intensity of the high-frequency modes of 39L-hBN/3LW in the range of 300–1300 cm^−1^ is almost identical to that of 3LW. This suggests that the interlayer EPC related to the C exciton of 3LW constituent in 39L-hBN/3LW is too weak to modify the intensity of the high-frequency intralayer modes of the constituents.

The rigid shear (S) and LB modes are the characteristic features of multilayer 2DMs^33,34^. Similar to multilayer graphene^33^, only the S_*N*,1_ (see Methods) modes are observed in *N*L-hBN (*N* > 1)^35^, while its LB modes are unobservable in the Raman spectra due to the Raman inactivity or weak EPC. In 3LW, one S mode (S_3,1_) and one LB mode (LB_3,2_) can be observed. Similar S mode with the same frequency is observed in 39L-hBN/3LW. It is assigned as the S_3,1_ mode of the 3LW constituent, being confirmed by its polarization Raman measurements^34^ (Fig. 1f). This indicates that the S mode of 39L-hBN/3LW is localized within the WS_2_ constituent because of the absence of overall in-plane restoring force, which can be ascribed to the lateral displacement induced by twist stacking and mismatched lattice constant (~20%) of the two layers at the twisted interface, similar to the S modes observed in various twisted 2DMs and vdWHs^36–40^. In contrast to the S mode, the corresponding LB_3,2_ mode of the 3LW flake is not observed in 39L-hBN/3LW. Interestingly, additional LB modes emerge in the 39L-hBN/3LW (Fig. 1e), as confirmed by its polarization Raman measurements^34^. These additional LB modes become weak when *E*_ex_ is away from *E*_C_ (Supplementary Fig. 2), indicating the LB modes of 39L-hBN/3LW may be resonantly enhanced by electronic transitions related to the C exciton of the 3LW constituent in 39L-hBN/3LW. The appearance of additional LB modes in hBN/WS_2_ vdWHs implies that the reduced symmetry in vdWHs with twist stacking can render many LB modes Raman active, in contrast to many Raman-inactive or unobservable LB modes in most 2DMs due to the high symmetry and perfect stacking. Furthermore, the C exciton in WS_2_ constituent can help to enhance the Raman intensity of these LB modes.

### Assignment of each LB mode in hBN/WS_2_ vdWHs

**Fig. 2:**
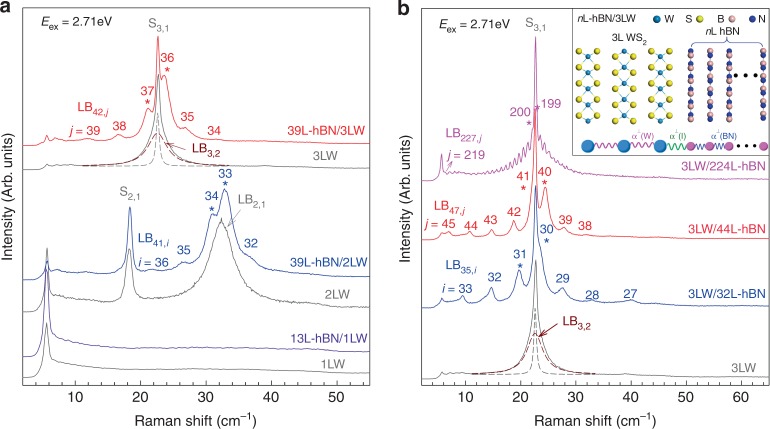
Raman spectra of hBN/WS_2_ and WS_2_/hBN with different numbers of WS_2_ and hBN layers in vdWHs. **a** Raman spectra of 13L-hBN/1LW (*θ*_t_ = 17.4°), 39L-hBN/2LW (*θ*_t_ = 0.3°) and 39L-hBN/3LW (*θ*_t_ = 0.3°) along with the corresponding WS_2_ flakes. The dark red and dash profile depicts the LB_3,2_ mode in 3LW while the gray dash profile represents the S_3,1_ mode. **b** Raman spectra of 3LW/32L-hBN (*θ*_t_ = 23.5°), 3LW/44L-hBN (*θ*_t_ = 23.5°) and 3LW/224L-hBN (*θ*_t_ = 17.2°) along with the standalone 3LW flake. The spectra are scaled and offset for clarify. The stars represent the two prime LB modes in vdWHs. The inset in **b** shows the schematic diagram of the linear chain model for the LB modes in *n*L-hBN/3LW

To figure out the physical origins of these emergent LB modes, we measured the Raman spectra of vdWHs with different numbers of WS_2_ and hBN layers in the constituents, as shown in Fig. 2. More LB modes are observed in hBN/WS_2_ vdWHs with MLW constituent than the corresponding standalone MLW flakes. The peak position of the LB modes (Pos(LB)) is significantly dependent on the number of layers in both WS_2_ (Fig. 2a) and hBN (Fig. 2b) constituents. However, no LB mode is observed in 1LW/13L-hBN.

The LB modes in 2DMs and vdWHs can be well described by the linear chain model (LCM)^33,34,36,37,40^. Here, we apply the LCM to assign the LB modes observed in hBN/WS_2_ vdWHs, as exemplified by *n*L-hBN/3LW vdWHs in the inset of Fig. 2b. The interlayer LB coupling of WS_2_ (*α*^⊥^(W) = 9.0 × 10^19^ Nm^−3^) is known from the previous experiments^41^, while that (*α*^⊥^(BN)) in hBN and the interfacial LB coupling (*α*^⊥^(I)) between hBN and WS_2_ constituents can be fitting parameters of the LCM. The experimental Pos(LB) of all *n*L-hBN/*m*LW ($m \, > \, 1$) in Fig. 2 can be well fitted by *α*^⊥^(BN) of 9.88 × 10^19^ Nm^−3^ and *α*^⊥^(I) of 8.97 × 10^19^ Nm^−3^. The fitted *α*^⊥^(BN) is in good agreement with the interlayer force constant of hBN^34^ based on inelastic X-ray scattering data, confirming the validity of the LCM. The mismatch of in-plane lattice constant between hBN and WS_2_ is about ~20%^42^, which is too large to form the moiré patterns dependent on twist angle (*θ*_t_). Therefore, in contrast to other vdWHs with *θ*_t_-dependent moiré patterns^38,39,43^, the *θ*_t_ is not so important to modify the interfacial LB coupling, similar to the case of MoS_2_/Graphene vdWHs^40^. Indeed, the frequency of observed LB modes in 13 samples of hBN/MLW or MLW/hBN vdWHs with twist angles of 0°–24°, among which four of them are hBN/MLW vdWHs and the others are MLW/hBN vdWHs, can be well reproduced by the same fitted *α*^⊥^(I). As exemplified in Supplementary Fig. 3, the LB modes observed in 3LW/44L-hBN (*θ*_t_ = 23.5°) and 43L-hBN/3LW (*θ*_t_ = 1.7°) exhibit slightly varied peak position, which can be well estimated by one fitted *α*^⊥^(I). This indicates that the interfacial LB coupling constant in hBN/WS_2_ vdWHs is independent on the stacking orders and twist angles. With the fitted *α*^⊥^(BN) and *α*^⊥^(I), Pos(LB) (in cm^−1^) and normal displacements of each LB mode in any *n*L-hBN/*m*LW can be further calculated (see Methods), which can be used to assign the observed LB modes in the *n*L-hBN/*m*LW, as shown in Fig. 2. The theoretically calculated evolution of Pos(LB) with number of layers of the hBN constituent (*n*) in *n*L-hBN/3LW is demonstrated in Supplementary Fig. 4a. The experimental Pos(LB) in *n*L-hBN/3LW or 3LW/*n*L-hBN (see Supplementary Fig. 4b) are also summarized in Supplementary Fig. 4a, which is in good agreement with the theoretical results. Interestingly, both Fig. 2 and Supplementary Fig. 4b show that only LB modes with Pos(LB) less than about 50 cm^−1^ are observed in hBN/WS_2_ vdWHs. Because *α*^⊥^(I) is comparable to *α*^⊥^(W) and *α*^⊥^(BN), the wavefunction of the LB phonons is well extended over the entire layers of the vdWHs, exhibiting bulk-like phonon features. The bulk manner of the LB phonons in layered material with larger than 10 layers can be further confirmed by their full width at half maximum, which approaches to that of the bulk material in 3D limit^34^. The interfacial coupling in hBN/WS_2_ vdWHs is so efficient that few-layer WS_2_ constituent can modify the layer displacements of the 3D LB modes of hBN constituent with 224-layer thickness to make more than 30 LB modes observable in 3LW/224L-hBN vdWHs, as shown in Fig. 2b. The peak positions of all these observed LB modes are in good agreement with the expected frequencies from LCM, further confirming that the LCM can well reproduce Pos(LB) of hBN/WS_2_ vdWHs with tens to hundreds of layer thickness.

### Peculiar resonance mechanism of LB modes in hBN/WS_2_ vdWHs

**Fig. 3:**
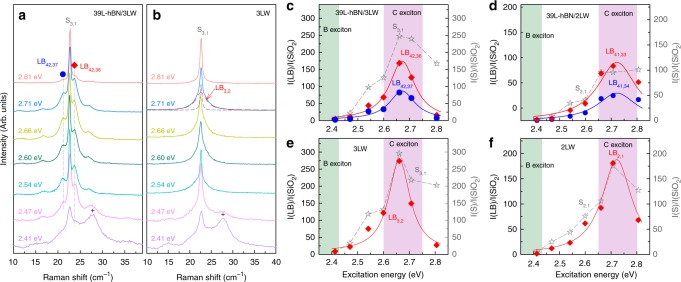
Intensity resonances of the S and LB modes in 39L-hBN/*m*LW and corresponding *m*LW. Raman spectra of **a** 39L-hBN/3LW and **b** 3LW excited by *E*_ex_ in the range of 2.41–2.81 eV. The diamonds and circles represent the two prime LB modes in 39L-hBN/3LW. The crosses show TA phonons resonant with the B exciton^45^. The resonant profiles of **c** the LB_42,36_ (red diamonds), LB_42,37_ (blue circles) and S_3,1_ (gray stars) in 39L-hBN/3LW, **d** the LB_41,33_ (red diamonds), LB_41,34_ (blue circles) and S_2,1_ (gray stars) modes in 39L-hBN/2LW, **e** the S_3,1_ (gray stars) and LB_3,2_ (red diamonds) modes in 3LW flake, **f** the S_2,1_ (gray stars) and LB_2,1_ (red diamonds) modes in 2LW flake. The diamonds, circles, and stars represent the experimental data while the red and blue solid lines are the fitting results. The Raman intensity is normalized by the *E*_1_ mode of quartz at ~127 cm^−1^ to eliminate the different efficiencies of charge-coupled device at different *E*_ex_

To further reveal the resonance mechanism of the interlayer Raman modes in hBN/WS_2_ vdWHs, seven *E*_ex_ are utilized to excite the Raman spectra in 39L-hBN/3LW and 39L-hBN/2LW and the corresponding WS_2_ flakes, as shown in Fig. 3a, b and Supplementary Fig. 5. The corresponding resonant profiles of the S and LB modes are depicted in Fig. 3c–f. The Raman intensities of the S and LB modes in both 39L-hBN/3LW and 39L-hBN/2LW exhibit a strong enhancement when *E*_ex_ matches *E*_C_ of the 3LW and 2LW flakes, respectively. The S and LB modes of the corresponding standalone WS_2_ flakes exhibit similar resonant behavior (Fig. 3e, f).

In principle, the resonant behaviors of the S and LB modes with a transition energy of *E*_sys_ in vdWHs can be described by the three-step Raman scattering process^44^:

1 $$I \propto \left| {\mathop {\sum}\limits_{e_{1},e^{\prime}_{1}} \frac{{\langle h|H_{{\mathrm{e - pht}}}|e_{1}^\prime \rangle \langle e_{1}^\prime |H_{{\mathrm{e - phn}}}|e_{1}\rangle \langle e_{1}|H_{{\mathrm{e - pht}}}|h\rangle }}{{(E_{{\mathrm{ex}}} - E_{{\mathrm{sys}}} - {\mathrm{i}}\gamma ){\kern 1pt} (E_{{\mathrm{ex}}} - E_{{\mathrm{ph}}} - E_{{\mathrm{sys}}} - {\mathrm{i}}\gamma )}}} \right|^2$$

where *e*_1_, $e_1^\prime$ and *h* are electron and hole states forming in the resonance Raman process, $\langle e_1|H_{{\mathrm{e - pht}}}|h\rangle$ and $\langle h|H_{{\mathrm{e - pht}}}|e_1^\prime \rangle$ correspond to the electron-photon interaction of the photon absorption and emission, respectively, $\langle e_1^\prime |H_{{\mathrm{e - phn}}}|e_1\rangle$ represents the EPC, *γ* is the resonance window width related to the lifetime for the Raman scattering process. The band structures of hBN/WS_2_ vdWHs are calculated by density functional theory calculation, as depicted in Supplementary Fig. 6. According to the band alignment, hBN/WS_2_ vdWHs correspond to type-I heterojunction. Because *E*_C_ of the standalone WS_2_ flake is much lower than the band gap of hBN, the electronic states in hBN/WS_2_ vdWHs corresponding to the C exciton of the WS_2_ flakes are well confined to the few-layer WS_2_ constituent of the vdWHs, exhibiting layer-localized 2D electronic states. The *E*_C_ in hBN/WS_2_ vdWHs is slightly different from that in the WS_2_ flakes due to the dielectric effect. The S modes in hBN/WS_2_ vdWHs are localized within the WS_2_ constituents due to the weak interfacial shear coupling between hBN and WS_2_ constituents. When *E*_ex_ approaches *E*_C_ of the WS_2_ flake, these localized S modes in vdWHs are greatly enhanced in intensity, similar to the case of the S modes in the corresponding standalone WS_2_ flake.

The resonant behavior of the two LB modes in 39L-hBN/3LW and 39L-hBN/2LW can be fitted by Eq. 1 with fitted parameter sets of (*E*_sys_ = 2.67 eV, *γ* = 0.06 eV) and (*E*_sys_ = 2.72 eV, *γ* = 0.09 eV), respectively. The fitted *E*_sys_ for the LB modes in the two vdWHs matches the *E*_C_ fitted from the LB modes in the corresponding standalone WS_2_ flakes (Fig. 3e, f), which are also in good agreement with those measured by reflectance contrast spectra^45^. As discussed above, the LB modes in hBN/WS_2_ vdWHs are bulk-like phonon modes extended over the entire layers of the vdWHs while the electronic states related to the C exciton are layer-localized 2D electronic states confined within the few-layer WS_2_ constituents. Therefore, the strong intensity resonance of the LB modes with *E*_C_ suggests the presence of an unusual and strong coupling between 2D electrons confined to its few-layer constituent and bulk-like 3D phonons extended over the entire vdWHs with tens to hundreds of layers, denoted as the constituent-vdWH EPC.

### Constituent-vdWH EPC mediated by interfacial coupling

Below we establish a microscopic picture of this unusual constituent-vdWH EPC to understand the relative intensity of the LB modes in hBN/*m*LW vdWHs ($m \, > \, 1$). In a first-order Raman scattering, the excitation photons with energy approaching *E*_C_ of the *m*LW flake can be strongly absorbed by the *m*LW constituent in hBN/*m*LW vdWHs to generate the electrons and holes related to the C exciton in the *m*LW constituent, which can be coupled with the LB vibrations within *m*LW constituent, similar to the case of a standalone *m*LW flake. Because of the strong interfacial LB coupling between the hBN and *m*LW constituents, these interlayer vibrations excited by the photons can efficiently interact with those in the hBN constituent, resulting in bulk-like collective LB vibrations of entire layers in the vdWHs. Therefore, the interfacial LB coupling between the hBN and *m*LW constituents mediates the so-called constituent-vdWH EPC in hBN/*m*LW vdWHs.

According to the empirical bond polarizability model, EPC is related to the change of bond polarizability, which is, to the first order, a function of the vibration displacements^44^. The extended interlayer bond polarizability model had also been proposed for 2DMs to well understand the peak intensity of the LB modes in few layer 2DMs^34,46^. Because the frequency difference between the observed LB modes in vdWHs is very small relative to *E*_ex_, the relative intensity between the LB modes in vdWHs is determined by their EPC strength. The LB modes in vdWHs exhibit a bulk-like collective vibrations of entire layers in the vdWHs through the interfacial LB coupling; thus, the interlayer displacements of certain LB mode in vdWH have a weighting factor of that from each LB mode in the *m*LW flakes. Because the laser excitation is directly resonant with *E*_C_ of *m*LW constituents in vdWHs, the EPC strength of a vdWH LB phonon can be estimated to be the sum of its weighting factor of interlayer displacements from all the LB modes in the standalone *m*LW flakes. The weighting factor of each LB mode in hBN/*m*LW vdWHs is given by the projection between its wavefunction component ($\psi$) (i.e., normal mode displacements) among the *m*LW constituents (see Methods) and the wavefunction ($\varphi _j$) of the $LB_{m,m - j}$ phonons ($j = 1,2, \ldots ,m - 1$) in standalone *m*LW flakes, i.e., $p_j = |\langle \varphi _j|\psi \rangle |$. The Raman intensity of the LB mode in hBN/*m*LW vdWHs is thus proportional to $p^2 = \mathop {\sum}\nolimits_j {\rho _jp_j^2}$ according to Eq. 1, where $\rho _j$ is the relative Raman intensity of the corresponding $LB_{m,m - j}$ mode in the standalone *m*LW flake. If the $LB_{m,m - j}$ mode is Raman inactive and can not be observed in the Raman spectrum, the corresponding $\rho _j$ is zero.

**Fig. 4:**
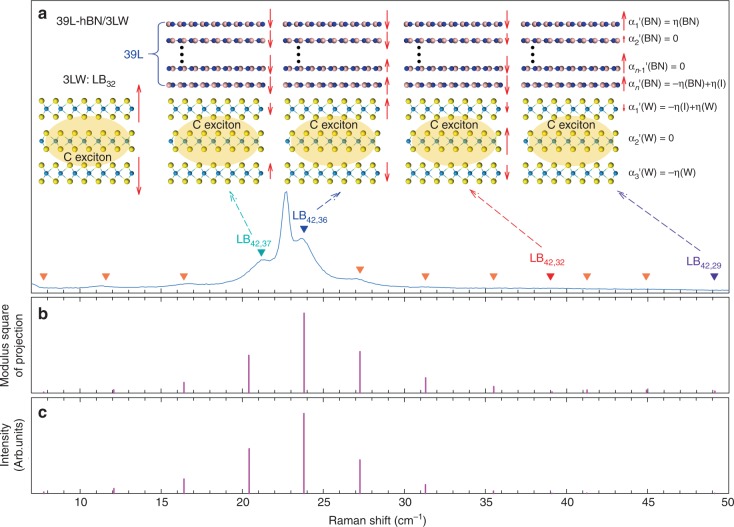
Schematic diagram for constituent-vdWH EPC of the LB modes in hBN/WS_2_ vdWHs. **a** Raman spectrum of a 39L-hBN/3LW in the region of 5~50 cm^−1^ and the normal mode displacements (red arrows) of the LB_42,37_, LB_42,36_, LB_42,32_, and LB_42,29_ modes in a 39L-hBN/3LW and the LB_3,2_ in a standalone 3LW flake. The triangles represent the representative expected LB modes in the 39L-hBN/3LW based on the LCM. In the *n*L-hBN/3LW (*n* = 39), $\alpha _i^\prime$(BN) (*i* = 1, 2, …, *n*) and $\alpha _j^\prime$(W) (*j* = 1, 2, 3) are the polarizability derivative of the entire layer *i* from hBN constituents and layer *j* from WS_2_ constituents with respective to the displacement in the *z* direction. **b** The modulus square of the projection from wavefunction of different LB modes in 39L-hBN/3LW vdWH onto the wavefunction of the LB_3,2_ mode in a standalone 3LW flake. **c** The relative intensity of LB modes in 39L-hBN/3LW vdWH based on the interlayer bond polarizability model

We take 39L-hBN/3LW as an example to illustrate the above microscopic picture. In the 3LW, the LB_3,1_ mode is Raman inactive and absent in the Raman spectrum; thus, only the LB_3,2_ mode is considered to calculate the Raman intensity of the LB mode in 39L-hBN/3LW vdWH. The interlayer displacements of the LB modes in 39L-hBN/3LW and the LB_3,2_ mode in a standalone 3LW are illustrated in Fig. 4a. The wavefunction $\varphi _1$ of the LB_3,2_ mode in the standalone 3LW flake is (1/$\sqrt 2$, 0, −1/$\sqrt 2$)^*T*^. The calculated *p*^2^ for different LB modes in the 39L-hBN/3LW vdWH are shown in Fig. 4b. The relative values of *p*^2^ for different LB modes in 39L-hBN/3LW vdWH are in good agreement with the relative intensity of the corresponding LB modes in the range of 5–50 cm^−1^ (Fig. 4a). Notably, because the three layers in the 3LW constituent of 39L-hBN/3LW vdWH are almost motionless for the LB_42,*i*_ (*i* = 1,2, …, 29) modes in the range of 50–120 cm^−1^, the phonon wavefunction projection from these modes in the 39L-hBN/3LW vdWH onto the LB_3,2_ mode in 3LW flake should be approximately zero. Thus, the absence of the LB modes with frequency larger than 50 cm^−1^ can be ascribed to the fact that such LB modes in vdWH does not involve the LB displacements of the layers in the WS_2_ constituent, and hence there is no coupling to the C exciton therein. Similar analysis can be applied to other hBN/MLW vdWHs, as 39L-hBN/2LW and 3LW/224L-hBN vdWHs shown in Supplementary Figs. 7 and 8, respectively. The good agreement between the Raman intensity and calculated *p*^2^ for different LB modes in hBN/WS_2_ vdWHs with tens to hundreds of layer thickness provides strong support for the above microscopic picture of the peculiar cross-dimensional coupling between the bulk-like 3D phonons extended over the entire vdWHs and the 2D electrons confined within the few-layer constituent.

### Interlayer bond polarizability model for LB modes in vdWHs

In principle, the relative Raman intensity of the LB modes in hBN/MLW vdWHs can also be directly calculated by the interlayer bond polarizability model^46^, in which each layer is simplified as a single object and the Raman intensity is related to the interlayer bond polarizability and bond vector. The total change of system’s polarizability by the interlayer vibration is the sum of the changes of each layer, ${\mathrm{\Delta }}\alpha = \mathop {\sum}\nolimits_i {\alpha _i^\prime \cdot {\mathrm{\Delta }}z_i}$, where $\alpha _i^\prime$ is the polarizability derivative of the entire layer *i* with respective to the displacement in the direction perpendicular to the plane (*z* direction) and Δ*z*_*i*_ is the normal displacement of layer *i* calculated from LCM for one given LB mode. The Raman intensity of this LB mode is proportional to |Δ*α*|^2^. As exemplified by *n*L-hBN/3LW (*n* = 39) in Fig. 4a, $\alpha _i^\prime$(BN) (*i* = 1, 2, …, *n*), and $\alpha _j^\prime$(W) (*j* = 1, 2, 3) assume simple values related to the polarizability derivative of the entire layer *i* in the hBN constituent and entire layer *j* in the WS_2_ constituent, respectively. According to the interlayer bond polarizability model^46^, only the top and bottom layers of hBN and WS_2_ constituents have non-zero values because they either have only one neighboring layer or have two non-equivalent neighboring layers at the interface between hBN and WS_2_ constituents (Fig. 4a), while the interior layers have zero values due to the cancellation effect from the two equivalent neighboring layers. Thus, we can simply denote $\alpha _1^\prime$(BN) = *η*(BN), $\alpha _n^\prime$(BN) = −*η*(BN) + *η*(I), $\alpha _1^\prime$(W) = −*η*(I) + *η*(W) and $\alpha _3^\prime$(W) = −*η*(W), where *η*(BN), *η*(W), and *η*(I) are fitting parameters related to the properties of the interlayer bond in hBN, WS_2_ constituents and that at the interface, respectively, including the normalized interlayer bond vector, interlayer bond length, interlayer bond polarizability, and their radial derivatives. Please note that *η*(W), *η*(I), and *η*(BN) depend on *E*_ex_, just like Raman intensities, and *η*(W) reaches the maximum when *E*_ex_ is resonant with *E*_C_. Thus, the change of *n*L-hBN/3LW polarizability is

2 $$\begin{array}{*{20}{l}} {{\mathrm{\Delta }}\alpha } \hfill & = \hfill & {\mathop {\sum}\limits_i {\kern 1pt} \alpha _i^\prime \cdot {\mathrm{\Delta }}z_i} \hfill \\ {} \hfill & = \hfill & {\eta (W)[{\mathrm{\Delta }}z_1(W) - {\mathrm{\Delta }}z_3(W)]} \hfill \\ {} \hfill & {} \hfill & { + \,\eta (BN)[{\mathrm{\Delta }}z_1(BN) - {\mathrm{\Delta }}z_n(BN)]} \hfill \\ {} \hfill & {} \hfill & { + \,\eta (I)[{\mathrm{\Delta }}z_n(BN) - {\mathrm{\Delta }}z_1(W)],} \hfill \end{array}$$

where the first term corresponds to the polarizability change from the WS_2_ constituent, the second term corresponds to the polarizability change from the hBN constituent and the third term corresponds to the polarizability change arising from the interface between WS_2_ and hBN constituents. Based on the Eq. 2 and the normal displacements available from the LCM, the relative Raman intensity of LB modes in 39L-hBN/3LW can be well fitted with *η*(I)/*η*(W) = 0.3 and *η*(BN)/*η*(I) = 0.003. This indicates that *η*(I) at the interface is comparable to *η*(W). However, since hBN is an insulator, *η*(BN) can be negligible compared with both *η*(I) and *η*(W), particularly under visible laser excitation. The corresponding calculated results of the relative Raman intensity of the LB modes in 39L-hBN/3LW are depicted in Fig. 4c, which is in line with the experimental results and also the calculated results according to the modulus square of the projection of phonon wavefunction. In Eq. 2, the first term dominates Raman intensity of a LB mode in the 39L-hBN/3LW, with a result that the larger the phonon wavefunction projection from the LB modes in the 39L-hBN/3LW onto the LB_3,2_ mode in 3LW flake is, the stronger the Raman intensity of the LB mode in the vdWH can be. Similar analysis based on the interlayer bond polarizability model can be applied to calculate the Raman intensity of the LB modes in other hBN/WS_2_ vdWHs, for example, 39L-hBN/2LW and 3LW/224L-hBN, as shown in Supplementary Fig. 9.

Finally, the change of interlayer bond polarizability in *n*L-hBN/1LW is shown in Supplementary Fig. 10a. There is no longer any interlayer bond from the 1LW constituent and the change of vdWH’s polarizability is Δ*α* = *η*(BN)[Δ*z*_1_(BN)-Δ*z*_*n*_(BN)] + *η*(I)[Δ*z*_*n*_(BN)-Δ*z*_1_(W)]. Due to *η*(I) = 0.3*η*(W), the Raman intensity of the LB phonons in 1LW/*n*L-hBN should be ~9% of the strongest LB mode in MLW/*n*L-hBN, too weak to be detected even when *E*_ex_ approaches *E*_C_ of the standalone 1LW flake, which is in consistent with the experimental results (see Supplementary Fig. 10b).

## Discussion

The models presented above can be generalized and extended to other vdWHs. The excellent agreement of the LB mode intensity between the theory and experimental data in *n*L-hBN/*m*LW vdWHs confirms that the Raman enhancement of the LB modes in hBN/WS_2_ vdWHs is from the constituent-vdWH EPC mediated by the interfacial coupling between hBN and WS_2_ constituents. This extraordinary constituent-vdWH EPC in hBN/WS_2_ vdWHs exhibits cross-dimensional features, which can be also generally present in various vdWHs formed by multilayer TMD constituents, such as graphene/WS_2_, hBN/MoS_2_, and hBN/WSe_2_. The observed LB modes in graphene/MoS_2_ vdWHs^40^ provide evidence of the generality of this cross-dimensional EPC. In these vdWHs, only the LB modes with frequency lower than ~60 cm^−1^ can be detected, and no LB modes can be observed in vdWHs stacked by monolayer MoS_2_ and multilayer graphene^40^. The reduced symmetry of vdWH renders many vdWH LB phonons to be Raman active while the exciton transition in its multilayer TMD constituents can greatly enhance the Raman intensity of these LB modes. Thus, this work shows a feasible approach to enhance Raman signals of normally weak LB modes in the constituent and enable observations of many branches of LB modes in vdWHs. In addition, it allows an effective way to study interfacial coupling and EPC in vdWHs. Furthermore, because the interfacial coupling mediated constituent-vdWH EPC is sensitive to the interfacial coupling in vdWHs and also the characteristics of the constituents, the Raman intensities of the LB modes in vdWHs can exhibit different tendencies depending on the details of the vdWHs. This provides a way to manipulate both the designable phonon excitations in vdWHs and their coupling to the electronic states by varying the constituents and engineering the interface, leading to various opportunities to explore electron-phonon interactions in vdWHs.

## Methods

### Sample preparation

The WS_2_ and hBN crystals were purchased from HQ Graphene (Netherlands). Poly(methyl methacrylate) (PMMA, Mw = 996,000 g mol^−1^) and anhydrous dichloromethane (DCM) were purchased from Sigma (Shanghai, China). Polydimethylsiloxane (PDMS) was purchased from Dow Corning (Midland, MI, USA). The WS_2_ and hBN flakes were firstly deposited on 90 nm SiO_2_/Si substrates by mechanical exfoliation method. The number of layers (or thickness) of hBN flakes can be measured by atomic force microscope (AFM) while that of WS_2_ can be identified by the interlayer Raman modes^34^. A drop of PMMA in DCM (3.0 wt%) was spin-coated on mechanically exfoliated hBN flakes on 90 nm SiO_2_/Si substrate at 3000 rpm, followed by covering a PDMS film with thickness of 1–2 mm to form the PDMS/PMMA/hBN hybrid structure. The PDMS/PMMA/hBN film was detached from the 90 nm SiO_2_/Si substrate with the aid of a small water droplet^47^. After that, the PDMS/PMMA/hBN film was stacked on top of WS_2_ flakes by using a micromanipulator under an optical microscope to form the PDMS/PMMA/hBN/WS_2_ hybrid structure. The PDMS film was easily peeled off from the PMMA/hBN/WS_2_ hybrid structure heated on a hot plate at 50 °C. Subsequently, the PMMA film on top of hBN/WS_2_ vdWHs was completely removed by washing in DCM on a hot plate at 50 °C. Thus, the hBN/WS_2_ vdWHs were left on 90 nm SiO_2_/Si substrate. Similarly, the WS_2_/hBN vdWHs were also prepared by this method. In order to enhance the interfacial interaction in vdWHs, the hBN/WS_2_ and WS_2_/hBN vdWHs were annealed at 300 °C in Ar atmosphere for 1 h^40^.

### Sample characterizations

Silicon nitride cantilever with the normal spring constant of 0.58 Nm^−1^ (DNP-S, Bruker) was used to obtain the atomic lattice images of hBN and WS_2_ flakes in contact mode by atomic force microscope (MultiMode8, Bruker) equipped with an E-scanner. The scanning angle of the tip is adjusted in order to obtain clear images with sample location fixed and the lattice orientations of WS_2_ and hBN relative to the scanning angle can be ascertained. The relative twist angle between WS_2_ and hBN constituents can be determined by the relative lattice orientations between these two constituents. All images were captured with a scan rate at 10–20 Hz and 256 × 256 pixel resolution and analyzed with the NanoScope Analysis software. The thickness of hBN flakes were measured by the atomic force microscope in tapping mode.

### Raman measurements

Raman spectra are measured at room temperature using a Jobin-Yvon HR-Evolution micro-Raman system equipped with a liquid-nitrogen-cooled charge couple detector (CCD), a ×100 objective lens (numerical aperture = 0.90). The excitation energies are 2.41, 2.47, 2.54, 2.60, 2.66, and 2.71 eV from Ar^+^ laser and 2.81 eV from He–Cd laser. The 3600 and 600 lines per mm gratings are used in the Raman measurements. The 3600 lines per mm grating enables each CCD pixel to cover 0.07 cm^−1^ at 2.54 eV. Plasma lines are removed from the laser lines via BragGrate bandpass filters and the Raman measurements down to 5 cm^−1^ for each excitation are enabled using three BragGrate notch filters with optical density of 3–4 and with full width at half-maximum of 5–10 cm^−1^. A typical laser power of ~0.1 mW is used to avoid sample heating.

### Linear chain model

The vdWHs must be considered as an overall system to model the LB modes in *n*L-hBN/*m*LW (*N* = *n* + *m*) vdWHs, where each rigid layer is considered as a ball with nearest-neighbor layer-breathing interaction, i.e., *α*^⊥^(W) and *α*^⊥^(BN) for interlayer coupling of WS_2_ and hBN, respectively, and *α*^⊥^(I) for the interfacial coupling between WS_2_ and hBN constituents. The frequency $\omega$ (in cm^−1^) and displacement patterns of the LB modes in *n*L-hBN/*m*LW can be calculated by solving the *N* × *N* (tridiagonal) linear homogenous equation:

3 $$\omega _i^2{\mathbf{Mu}}_{\mathbf{i}} = \frac{1}{{2\pi ^2c^2}}{\mathbf{Du}}_{\mathbf{i}}$$

which can be simplified as

4 $$\omega _{i}^2{\mathbf{u}}_{\mathbf{i}} = \frac{1}{{2\pi ^2c^2}}{\tilde{\mathbf{D}}}{\mathbf{u}}_{\mathbf{i}}$$

where **u**_**i**_ is the phonon eigenvector of the *i*th mode with frequency $\omega _i$, **M** is the diagonal mass matrix of *n*L-hBN/*m*LW vdWHs in which *M*_*ij*_ is the mass per unit area of each rigid layer, *c* = 3.0 × 10^10^ cm s^−1^ and **D** is the LB force constant matrix. ${\tilde{\mathbf{D}}}$ is the simplified LB force constant matrix, which can be represented by (taking 2L-hBN/2LM as an example)

5 $$\left( {\begin{array}{*{20}{c}} { - {\textstyle{{\alpha ^ \bot ({\mathrm{BN}})} \over {m({\mathrm{BN}})}}}} & {{\textstyle{{\alpha ^ \bot ({\mathrm{BN}})} \over {m({\mathrm{BN}})}}}} & 0 & 0 \\ {{\textstyle{{\alpha ^ \bot ({\mathrm{BN}})} \over {m({\mathrm{BN}})}}}} & { - {\textstyle{{(\alpha ^ \bot ({\mathrm{BN}}) \, + \, \alpha ^ \bot (I))} \over {m({\mathrm{BN}})}}}} & {{\textstyle{{\alpha ^ \bot (I)} \over {m({\mathrm{BN}})}}}} & 0 \\ 0 & {{\textstyle{{\alpha ^ \bot (I)} \over {m({\mathrm{W}})}}}} & { - {\textstyle{{(\alpha ^ \bot ({\mathrm{I}}) \, + \, \alpha ^ \bot ({\mathrm{W}}))} \over {m({\mathrm{W}})}}}} & {{\textstyle{{\alpha ^ \bot ({\mathrm{W}})} \over {m({\mathrm{W}})}}}} \\ 0 & 0 & {{\textstyle{{\alpha ^ \bot ({\mathrm{W}})} \over {m({\mathrm{W}})}}}} & { - {\textstyle{{\alpha ^ \bot ({\mathrm{W}})} \over {m({\mathrm{W}})}}}} \end{array}} \right)$$

where *m*(BN) and *m*(W) are the equivalent atomic mass pert unit area of monolayer hBN and monolayer WS_2_, respectively. The interlayer LB coupling of WS_2_ is known as *α*^⊥^(W) = 9.0 × 10^19^ Nm^−3^ from the previous work^41^. With the experimental frequencies of LB modes in 39L-hBN/3LW, 3LW/32L-hBN, and 3LW/44L-hBN shown in Fig. 2, the interlayer coupling within the hBN constituent (*α*^⊥^(BN)) and the interfacial coupling between hBN and WS_2_ constituents (*α*^⊥^(I)) can be estimated as *α*^⊥^(BN) = 9.88 × 10^19^ Nm^−3^ and *α*^⊥^(I) = 8.97 × 10^19^ Nm^−3^. Based on *α*^⊥^(W), *α*^⊥^(BN) and *α*^⊥^(I), Pos(LB) and the corresponding interlayer displacement patterns of each LB modes can be calculated, as shown in Fig. 4 and Supplementary Fig. 4.

For the isotropic 2DMs with layer number *N*, there are *N*-1 pairs of S and *N*-1 LB modes in isotropic 2DMs with layer number *N*, denoted as S_*N*,*N*−*j*_ and LB_*N*,*N*−*j*_ (*j* = *N*-1, *N*-2, …, 2, 1), respectively. S_*N*,1_ and LB_*N*,1_ correspond to the S and LB modes with highest frequency for each *N*, respectively. In *n*L-hBN/*m*LW (*N* = *n* + *m*) vdWHs, there are *N*-1 LB modes, which can be denoted as LB_*N*,*N*−*j*_ (*j* = *N*-1, *N*-2, …, 2, 1). LB_*N*,1_ corresponds to the LB mode with highest frequency for each *N*. In contrast, there are *m* pairs of S modes from WS_2_ constituents of the *n*L-hBN/*m*LW (*N* = *n* + *m*) vdWHs. We denote each S mode as S_*m*,*m*−*i*_ where *i* is the number of phonon branches and *i* = *m*-1, *m*-2, …, 2, 1. S_*m*,1_ corresponds to the S mode with highest frequency for each *m*.

### Density functional theory calculation

The band structure and density of states (DOS) of hBN/WS_2_ heterostructure are calculated by using the Vienna ab initio simulation package based on density functional theory^48–50^. The electron-ion interaction is described by the Projector Augmented Wave pseudopotentials. The exchange-correlation functional is described by Perderw, Burke, and Ernzerhof version of the generalized gradient approximation^51–53^. A plane-wave basis set with an energy cut-off of 400 eV was used in our calculations. The conjugated gradient method was proposed in the geometry optimization. The convergence condition for the energy is 10^−6^ eV and the structure were relaxed until the force on each atom was less than 0.05 eVÅ^−1^. Because of the lattice constant mismatch between hBN and WS_2_, 5 × 5 supercell of hBN and 4 × 4 supercell of WS_2_ were used to construct the composite supercell of hBN/WS_2_ heterostructure to minimize the atomic strain. The optimizated structure is subsequently used for electronic state calculations.

### Calculations for the phonon wavefunction projection

Taking the 39L-hBN/3LW vdWH as an example. As demonstrated above, by applying the LCM with interfacial LB coupling between hBN and WS_2_ constituents, the phonon wavefunction (normal mode displacements) of each LB mode in 39L-hBN/3LW vdWH can be calculated, as LB_42,37_, LB_42,36_, LB_42,32_, and LB_42,29_ depicted by the in Fig. 4a. For example, the phonon wavefunction ($\psi ^0$) of LB_42,37_ and LB_42,36_ are $\psi _{42,37}^0$ = (−0.214, −0.190, …, −0.215, −0.192, −0.129, 0.04, 0.172)^*T*^, $\psi _{42,36}^0$ = (−0.213, −0.180, …, 0.141, 0.194, 0.226, 0.015, −0.212)^*T*^, respectively. The components ($\psi$) of $\psi ^0$ among 3LW constituents is $\psi _{42,37}$ = (−0.129, 0.04, 0.172)^*T*^, $\psi _{42,37}$ = (0.226, 0.015, −0.212)^*T*^ for LB_42,37_ and LB_42,36_ in 39L-hBN/3LW vdWH, respectively. Similarly, the phonon wavefunction ($\varphi _j$) of the LB_*m*,*m*−*j*_ modes (*j* = 1, 2, …, *m*-1) in *m*LW flakes can also be obtained by the LCM. For example, for the Raman-active LB_3,2_ mode in a standalone 3LW, the phonon wavefunction is *φ*_1_ = (0.707, 0, −0.707)^*T*^. The EPC strength of a vdWH LB phonon can be estimated to be the sum of its weighting factor of interlayer displacements from all the LB modes in the standalone *m*LW flakes. The weighting factor of each LB mode in hBN/*m*LW vdWHs can be calculated by the modulus of inner product of $\psi$ and *φ*_*j*_, *i*.*e*., $p_j = |\langle \varphi _j|\psi \rangle |$. The Raman intensity of the LB mode in hBN/*m*LW vdWHs is thus proportional to $p^2 = \mathop {\sum}\nolimits_j {\rho _jp_j^2}$, where $\rho _j$ is the relative Raman intensity of the corresponding $LB_{m,m - j}$ mode in the standalone *m*LW flake.

## Supplementary information

Supplementary Information

## Data Availability

The data that support the findings of this study are available from the corresponding author upon reasonable request.
