# Supplementary material for: Cross-dimensional electron-phonon coupling in van der Waals heterostructures
Source: Nat Commun. 2019 Jun 3;10:2419. doi: 10.1038/s41467-019-10400-z (PMC6546732; doi:10.1038/s41467-019-10400-z)
Supplement: Supplementary file 1 — Supplementary Information [file 41467_2019_10400_MOESM1_ESM.pdf]

Cross-dimensional electron-phonon coupling in van der Waals heterostructures

Lin *et al.*

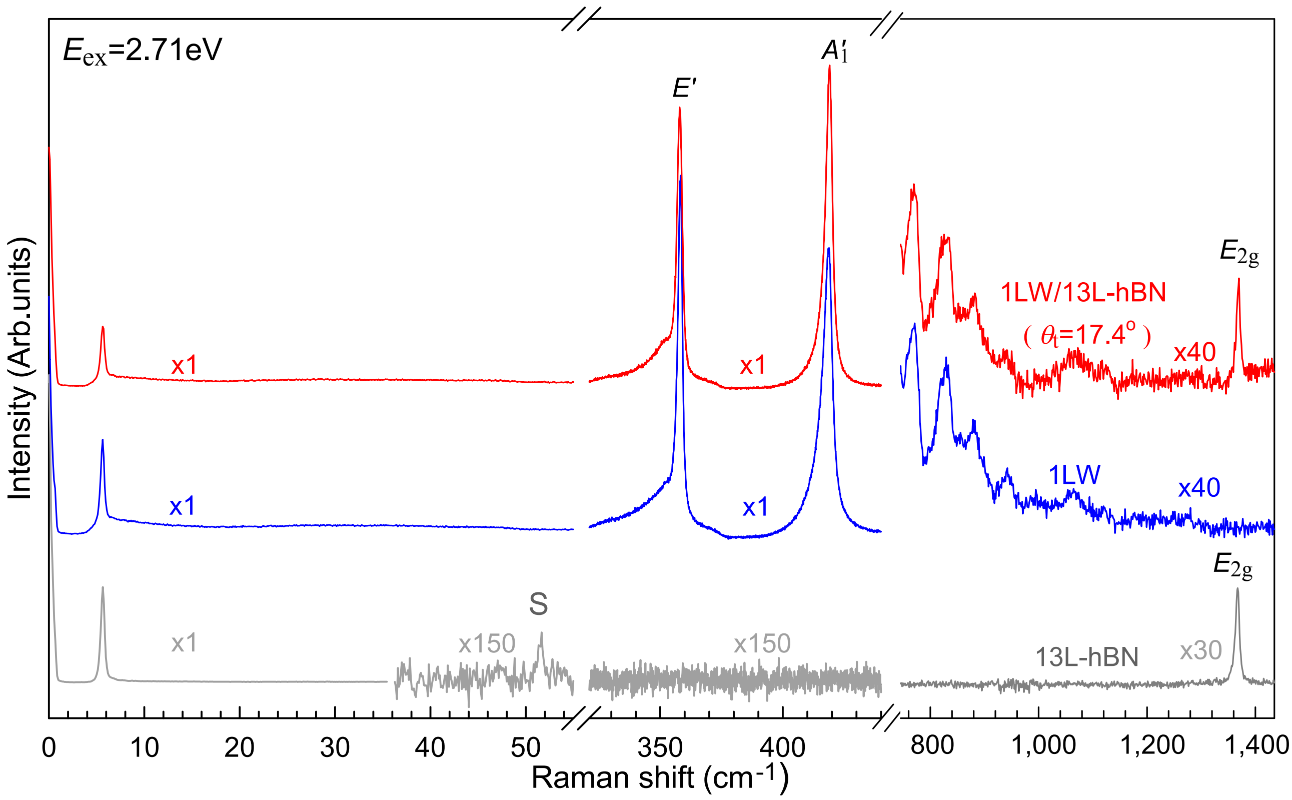

Supplementary Figure 1 | Raman spectra of hBN/WS_2 vdWHs and 1LW. Raman spectra of 13L-hBN, 1LW and 1LW/13L-hBN under excitation E_{ex}=2.71 eV. The spectra are scaled and offset for clarify and the scale factors are shown.

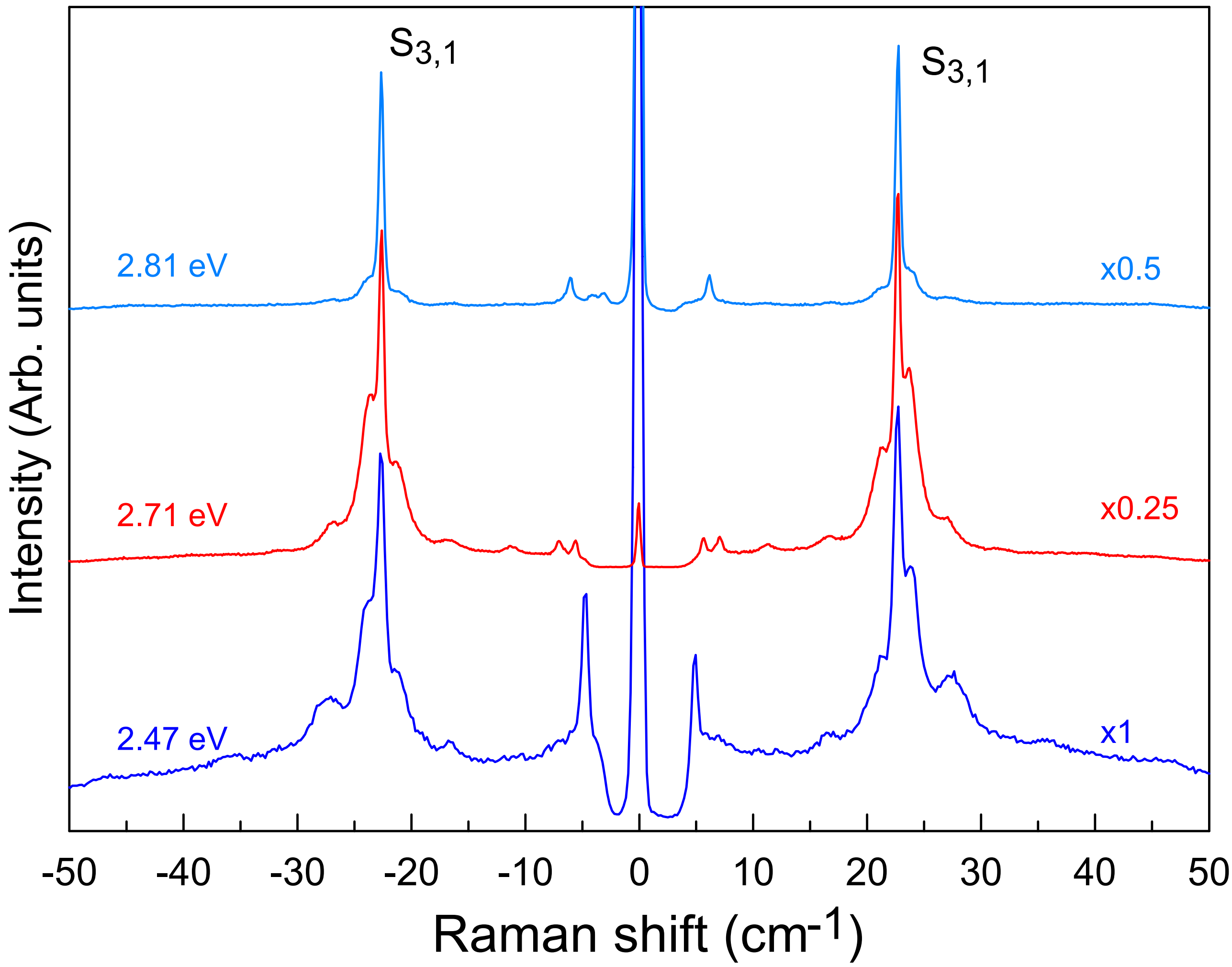

Supplementary Figure 2 | Resonance Raman spectra of 39L-hBN/3LW. Raman spectra of 39L-hBN/3LW excited by E_{ex}=2.41, 2.71, 2.81 eV. The spectra are scaled and offset for clarify and the scale factors are shown.

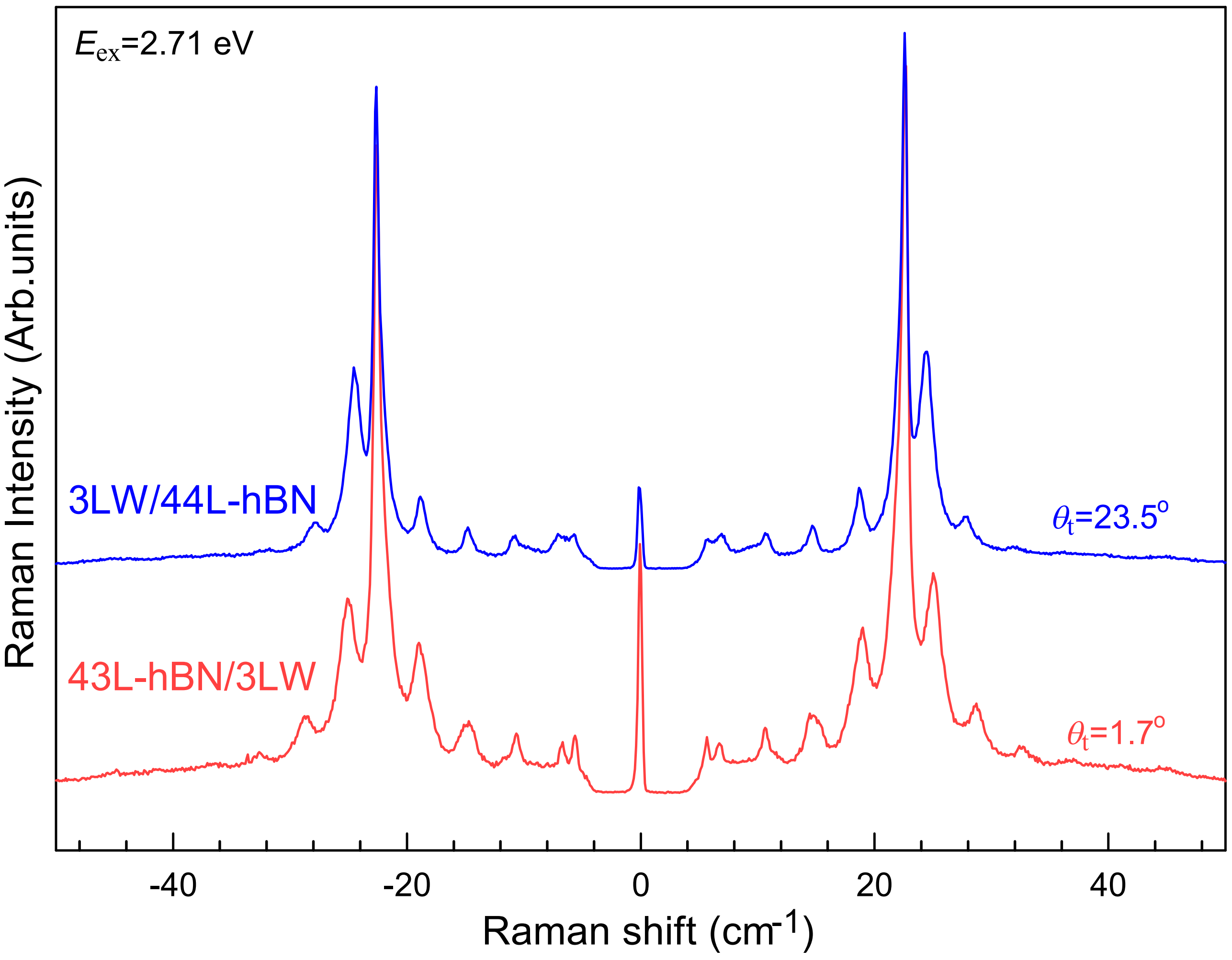

Supplementary Figure 3 | Raman spectra of hBN/MLW and MLW/hBN vdWHs. The ultralow-frequency Raman spectra of 3LW/44L-hBN (θ_t=23.5°) and 43L-hBN/3LW (θ_t=1.7°) excited by E_{ex} of 2.71 eV.

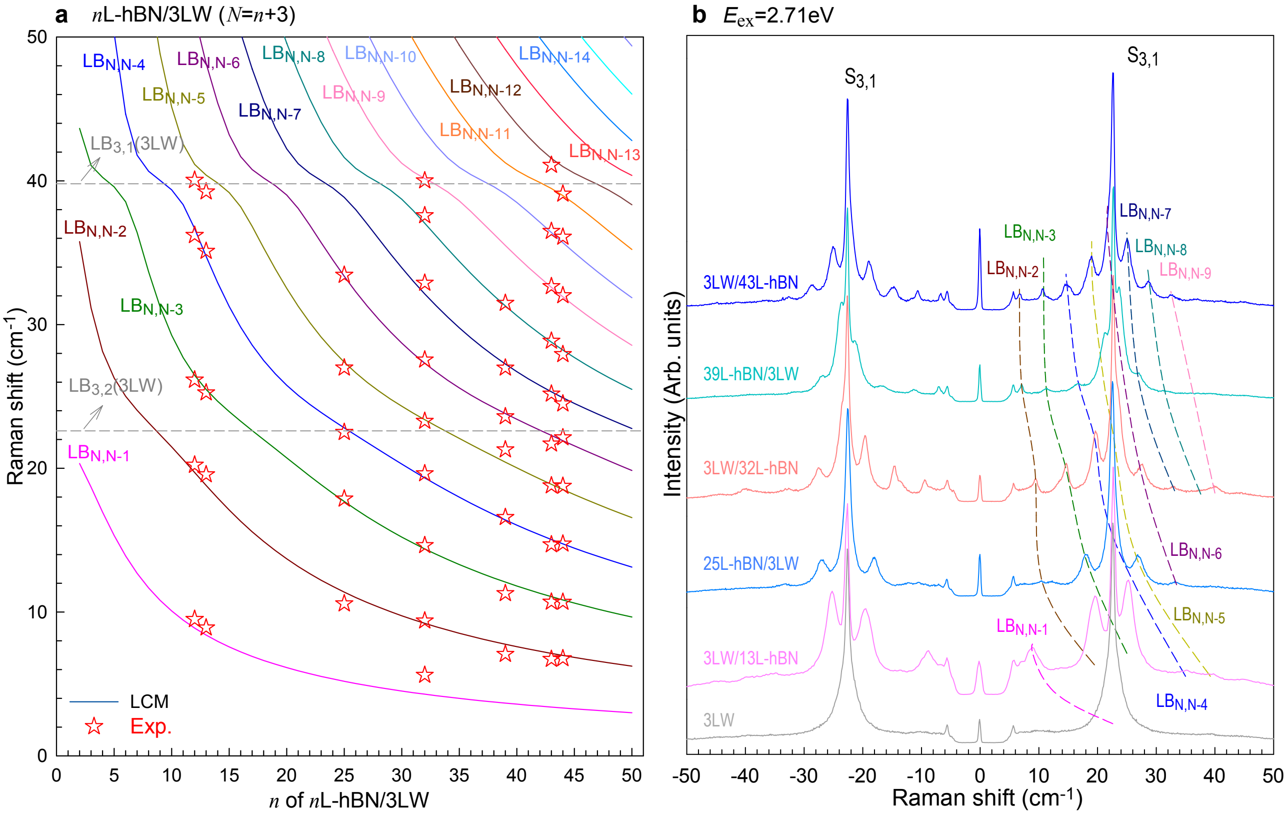

Supplementary Figure 4 | The frequencies of the LB modes in nL-hBN/3LW calculated from LCM. (**a**) Theoretical evolution of the LB modes in nL-hBN/3LW (n=1,2,...,50) as a function of the number n of layers in the hBN constituent. (**b**) Stokes/anti-Stokes Raman spectra of nL-hBN/3LW and 3LW/nL-hBN in the range of -50∼50 cm^{-1}. The corresponding experimental data (Exp.) of 3LW/nL-hBN are denoted as red stars.

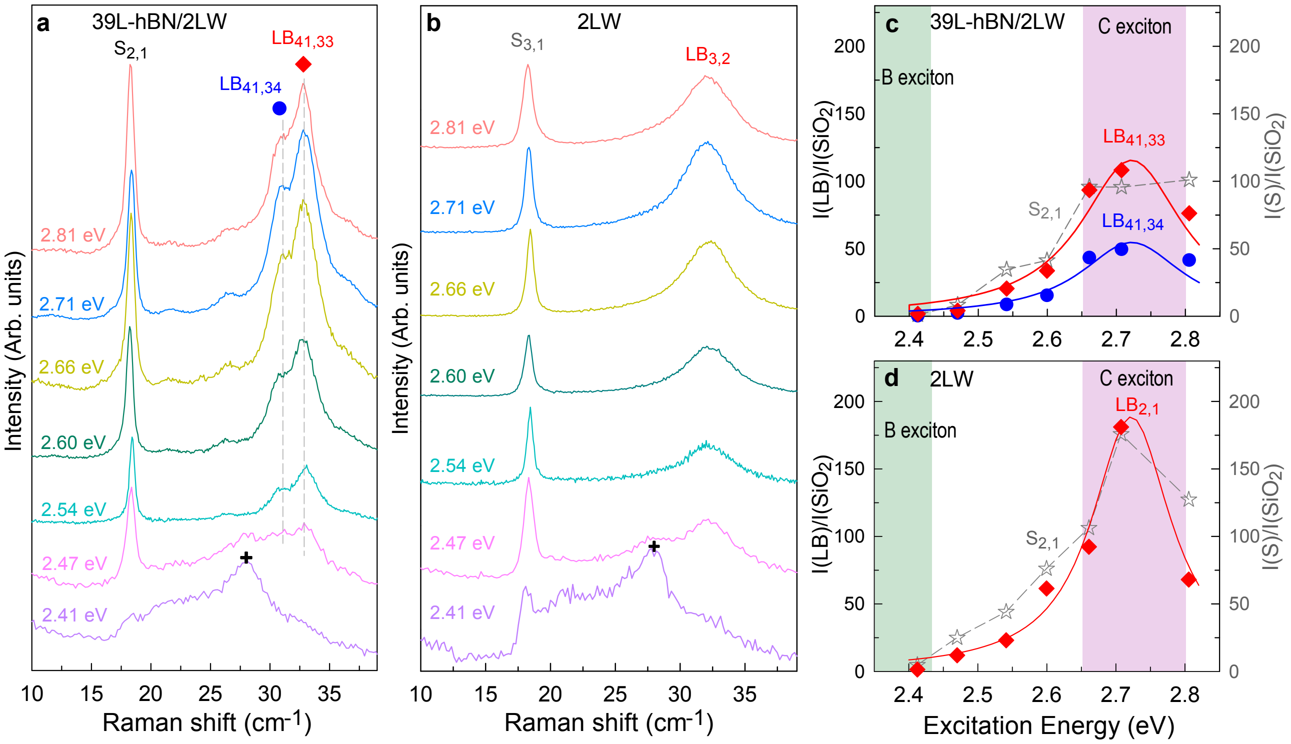

Supplementary Figure 5 | Intensity resonances of the S and LB modes in 39L-hBN/2LW and 2LW. Raman spectra of (**a**) 39L-hBN/2LW and (**b**) 2LW excited by E_{ex} in the range of 2.41-2.81 eV. The diamonds and circles represent the two prime LB modes in vdWH. The crosses show TA phonons of 2LW in resonance with the B exciton. The resonant profiles of (**c**) the $LB_{41,33}$ (red diamonds), $LB_{41,34}$ (blue circles) and $S_{2,1}$ (grey stars) modes in 39L-hBN/2LW, (**d**) the $S_{2,1}$ (grey stars) and $LB_{2,1}$ (red diamonds) modes in the standalone 2LW flake. The diamonds, circles and stars are the experimental data while the red and blue solid lines are the fitting results.

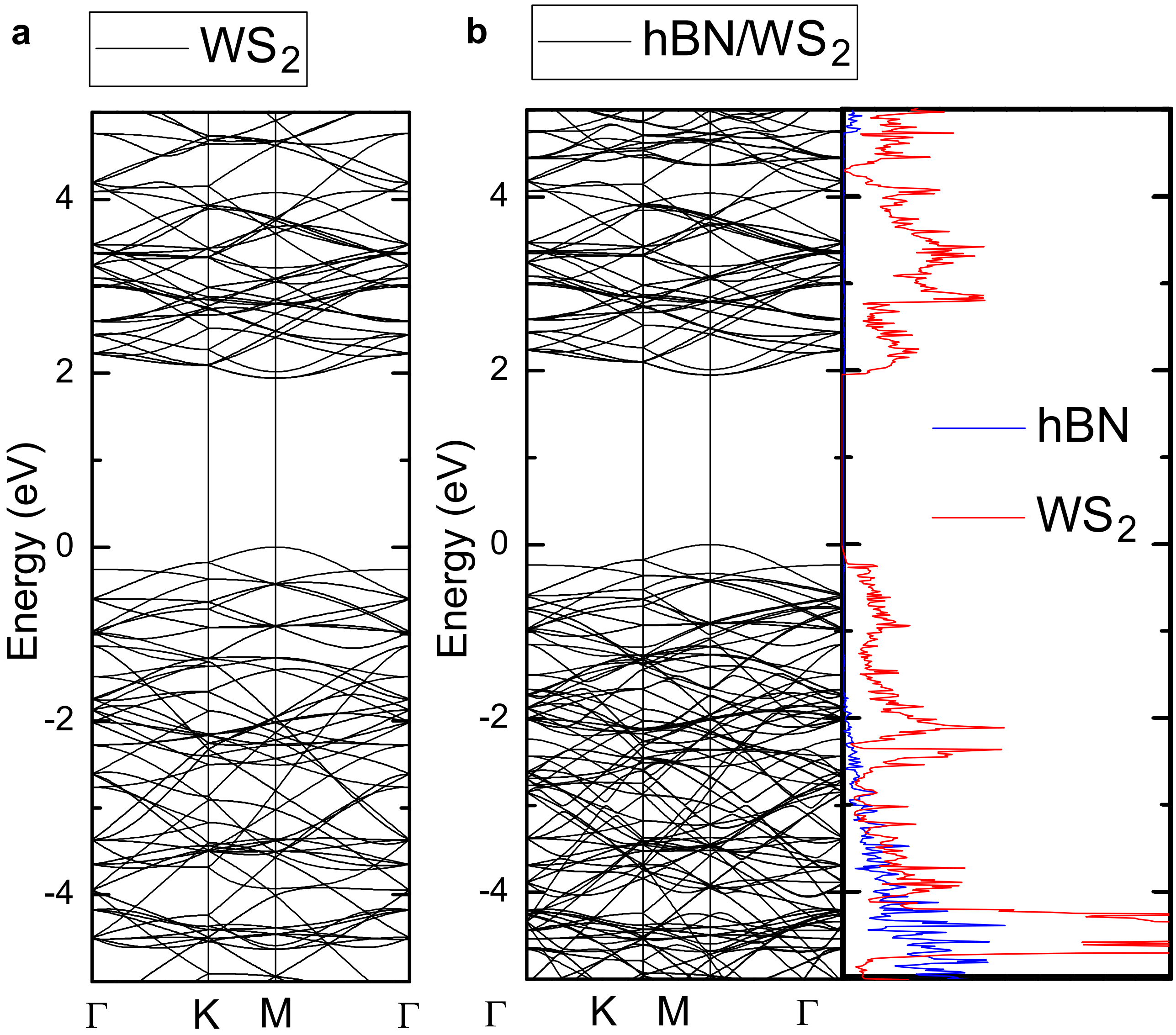

Supplementary Figure 6 | Band alignment of hBN/WS_2 vdWHs. Band structure of the (**a**) 1LW and (**b**) hBN/WS_2 vdWHs. The partial density of states from hBN and WS_2 constituents is also shown. The band alignment of hBN/WS_2 vdWH is classified into the Type-I structure.

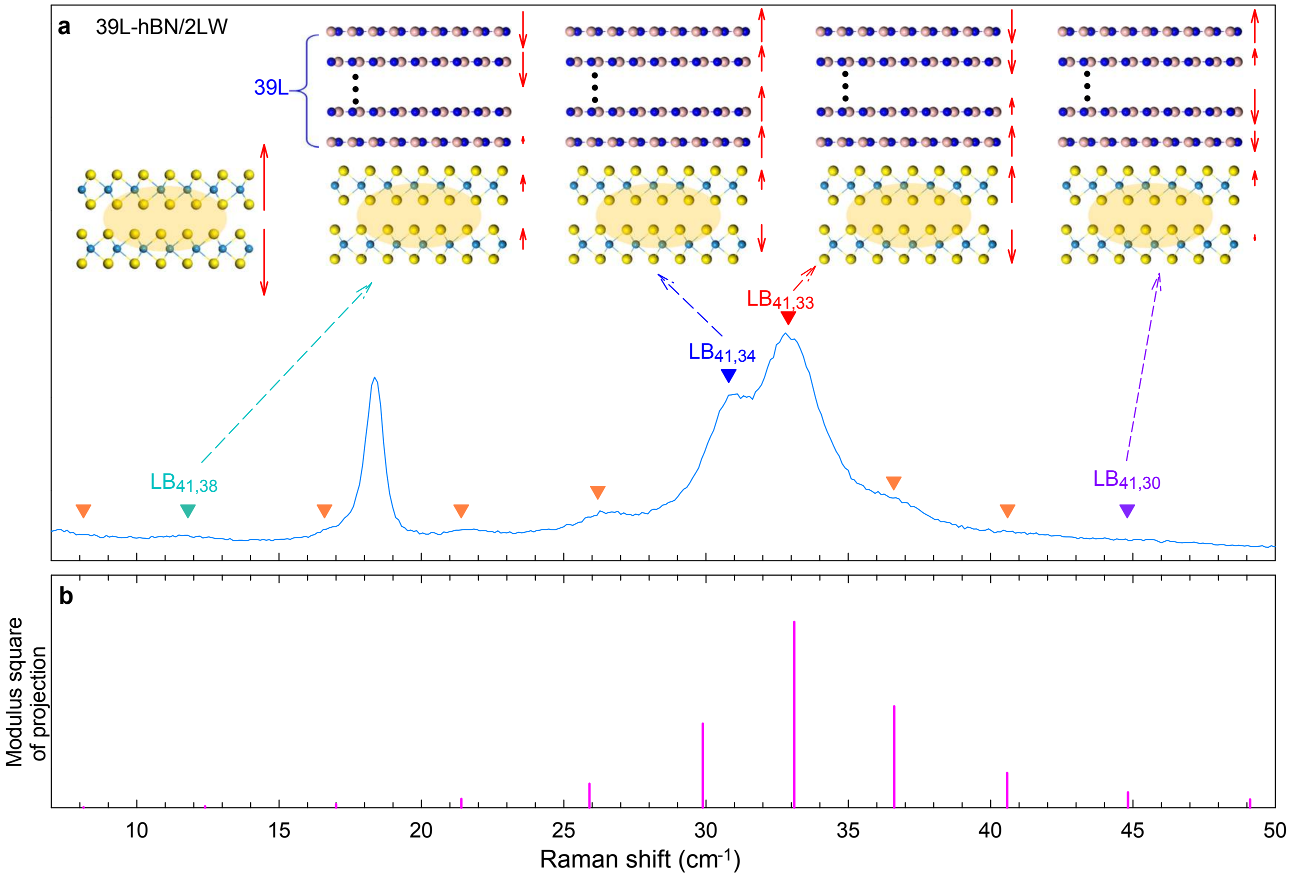

Supplementary Figure 7 | Schematic diagram for constituent-vdWH EPC of the LB modes in 39L-hBN/2LW vdWH. (**a**) Raman spectrum of 39L-hBN/2LW in the region of 5∼50 cm^{-1} and the normal mode displacements (red arrows) of the $LB_{41,38}$, $LB_{41,34}$, $LB_{41,33}$ and $LB_{41,30}$ modes in 39L-hBN/2LW and $LB_{2,1}$ in a standalone 2LW flake. The triangles represent the representative expected LB modes based on the LCM. (**b**) The modulus square of the projection from wavefunction of different LB modes in 39L-hBN/2LW vdWH onto that of the $LB_{2,1}$ mode in a standalone 2LW flake.

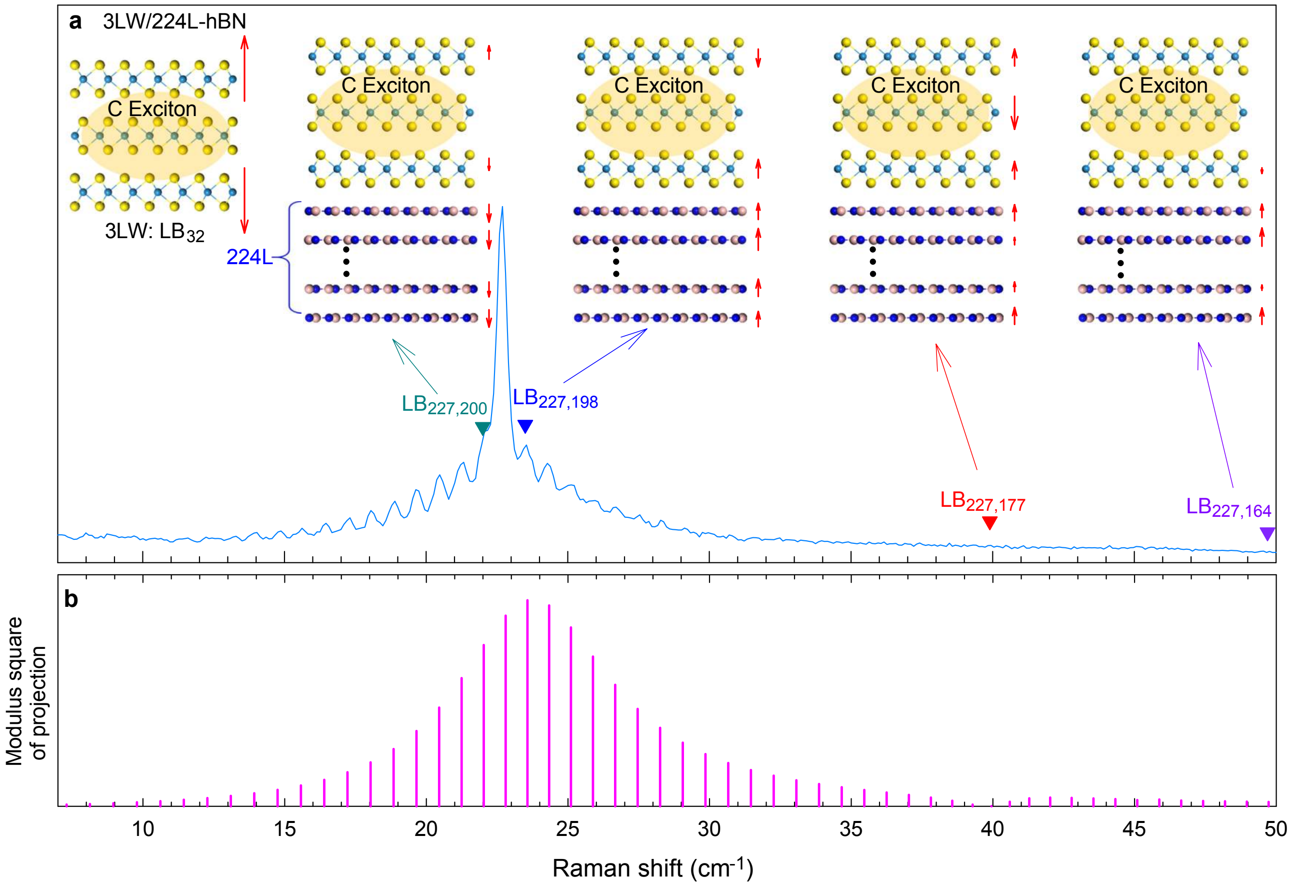

Supplementary Figure 8 | Schematic diagram for constituent-vdWH EPC of the LB modes in 3LW/224L-hBN vdWH. (**a**) Raman spectrum of 3LW/224L-hBN in the region of 5∼50 cm^{-1} and the normal mode displacements (red arrows) of the $LB_{227,200}$, $LB_{227,198}$, $LB_{227,177}$ and $LB_{227,164}$ modes in 3LW/224L-hBN and $LB_{3,2}$ in a standalone 3LW flake. The triangles represent the representative expected LB modes based on the LCM. (**b**) The modulus square of the projection from wavefunction of different LB modes in 3LW/224L-hBN vdWH onto that of the $LB_{3,2}$ mode in a standalone 3LW flake.

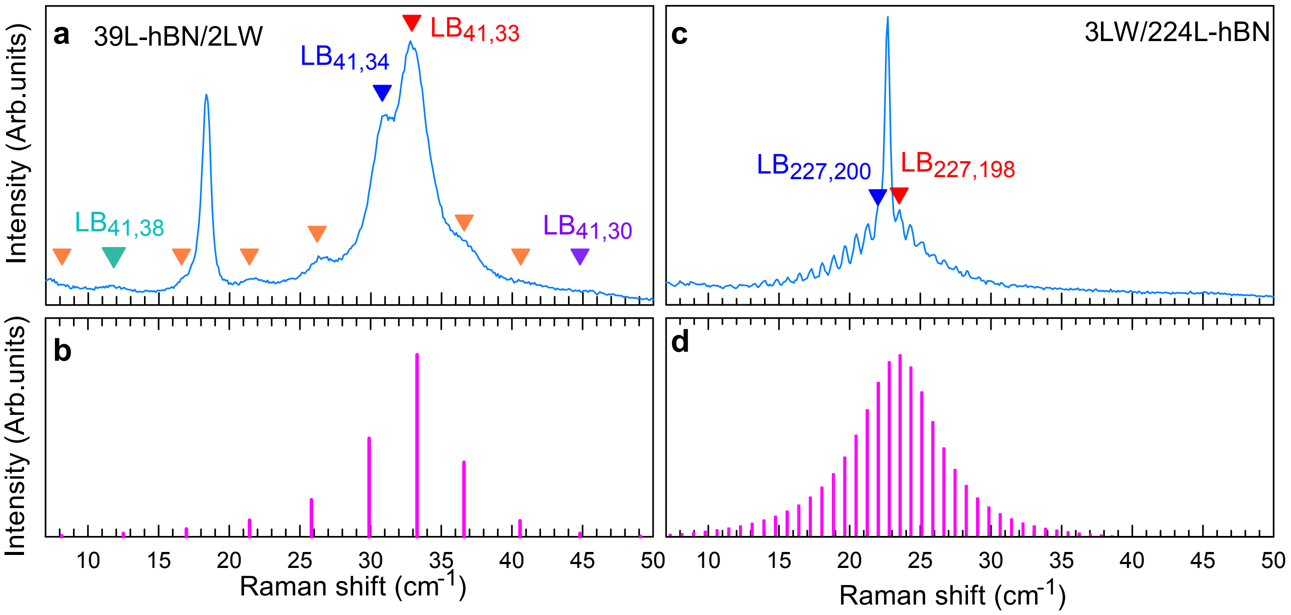

Supplementary Figure 9 | The Raman intensity of LB modes in hBN/WS_2 vdWHs based on the interlayer bond polarizability model. (**a**) Raman spectra of 39L-hBN/2LW. (**b**) The relative Raman intensity of the LB modes in 39L-hBN/2LW based on the interlayer bond polarizability model. (**c**) Raman spectra of 3LW/224L-hBN. (**d**) The relative Raman intensity of the LB modes in 3LW/224L-hBN based on the interlayer bond polarizability model. The triangles in **a** and **c** represent the representative expected LB modes based on the LCM.

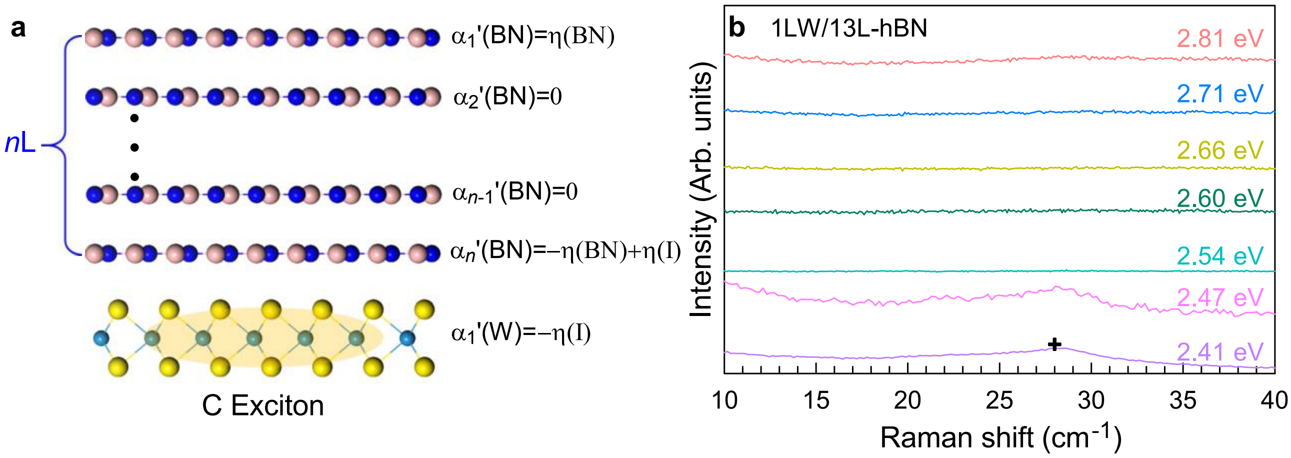

Supplementary Figure 10 | Interlayer bond polarizability model for 1LW/nL-hBN and Raman spectra of 1LW/13L-hBN. (**a**) The polarizability derivative for each stacking layer in z direction in 1LW/nL-hBN. α_i'(BN), and α_i'(W) are the polarizability derivative of the entire layer i with respective to the displacement in the z direction from hBN and WS_2 constituents, whose values can be derived from the interlayer bond polarizability and interlayer bond vectors and can be represented by η(BN) and η(I). η(BN) and η(I) are related to the properties of the interlayer bond, such as the interlayer bond length and polarizability derivatives, in hBN constituent and that at the interface, respectively. (**b**) Ultralow-frequency Raman spectra of 1LW/13L-hBN excited by E_{ex} in the range of 2.41-2.81 eV. The crosses shows TA phonon mode of 1LW in resonance with the B exciton.
